# Mechanotransduction of Collagen Biostimulators: Comparative Signaling and Implications for GLP‐1 RA–Associated Facial Aging

**DOI:** 10.1111/jocd.71105

**Published:** 2026-08-04

**Authors:** Chang‐Hwan Cho, Atsuko Yanagawa, Beomjoon Kim

**Affiliations:** ^1^ Dong‐An Joongsim Clinic Gangnam‐gu Seoul Republic of Korea; ^2^ Yanagawa Clinic Chuo‐ku Osaka Japan; ^3^ Institute of Industrial Science The University of Tokyo Meguro‐ku Tokyo Japan

**Keywords:** adipocyte, facial aging, glucagon‐like peptide‐1 receptor agonists, integrin, mechanotransduction, Piezo1, weight loss

## Abstract

**Background:**

GLP‐1 receptor agonists (GLP‐1 RAs) can induce rapid weight reduction, often associated with facial volume loss. However, optimal regenerative strategies remain unclear.

**Aims:**

To compare the mechanotransduction profiles of the major injectable collagen biostimulator classes in GLP‐1 RA–induced facial aging.

**Methods:**

A structured narrative review with Preferred Reporting Items for Systematic Reviews and Meta‐Analyses (PRISMA)‐informed search and reporting was conducted across PubMed, Embase, Web of Science, and Cochrane Library (January 1, 2000—March 31, 2026).

**Results:**

Forty‐three studies met the inclusion criteria. Each of the six biostimulator classes—PLLA, PDLLA, PDO, CaHA, particulate PCL, and liquid‐type PCL—exhibits a distinct mechanotransduction profile, though the strength of supporting evidence varies substantially across agents.

**Conclusions:**

Mechanotransduction‐active collagen biostimulators provide a biologically plausible rationale for addressing facial volume loss, although current clinical evidence remains limited and no studies have directly enrolled GLP‐1 RA–associated facial aging patients. A conceptual severity‐stratified framework warrants prospective validation.

## 1 Introduction

Semaglutide and tirzepatide are now widely used anti‐obesity agents globally, and next‐generation agents (retatrutide, cagrilintide/semaglutide [CagriSema], orforglipron) are expected to further expand the treated population [1, 2, 3]. Women constitute approximately 70% of users, with the majority aged 35–65 years [4]. Weight reductions of 15%–22% are routinely achieved [1, 2].

This therapeutic success has been associated with facial aging changes, sometimes referred to as “Ozempic Face,” a drug‐induced facial aging phenotype marked by rapid volume deflation, skin laxity, and premature skeletal appearance [5, 6]. Mechanobiologically, the condition represents a collapse of tensional homeostasis [7, 8]. In a healthy dermis, fibroblasts maintain an elongated morphology through integrin–focal adhesion kinase (FAK) complexes, Piezo1 channels, and YAP/TAZ, transducing mechanical input into procollagen transcription [9, 10, 11, 12]. When subcutaneous fat is rapidly depleted, this input collapses [7, 13]. Concurrently, GLP‐1 receptor stimulation has been reported to modulate macrophage polarization in a single review [6]; this observation requires confirmation in facial tissue contexts. Caloric deficit may plausibly contribute to facial muscle atrophy, by extrapolation from systemic sarcopenia data; however, no studies have directly examined facial musculature in GLP‐1 RA users [14, 15]. It should be noted that GLP‐1 RA may exert opposing influences on skin aging: while rapid fat loss accelerates facial aging, concurrent reductions in circulating advanced glycation end‐products may attenuate systemic inflammatory aging; whether these opposing mechanisms result in net skin benefit or harm remains unresolved. All clinical inferences in this population currently represent extrapolations.

Hyaluronic acid (HA) fillers function through passive space‐occupation; although newer cross‐linked HA formulations may elicit limited biomechanical responses, their low storage modulus (G′) may be insufficient to sustain mechanoreceptor activation [16, 17]. In contrast, collagen biostimulators form scaffolds capable of engaging mechanotransduction pathways [9, 10, 11, 12, 13, 18]. This review comparatively examines the mechanotransduction profiles of major biostimulator classes and discusses their potential—though clinically unvalidated—relevance in GLP‐1 RA–induced facial aging.

## 2 Materials and Methods

### 2.1 Study Design

This is a structured narrative review employing PRISMA‐informed search and reporting for transparency; it does not constitute a systematic review. The PRISMA flow diagram was used solely to document the study selection process and does not imply systematic review methodology. No formal risk‐of‐bias tool (e.g., ROBINS‐I, RoB 2.0) or GRADE certainty assessment was applied. The screening model (one primary screener with 20% cross‐verification) did not meet the dual‐independent criterion required for systematic reviews. The protocol was not registered with PROSPERO but is available upon request.

### 2.2 Search Strategy

PubMed/MEDLINE, Embase, Web of Science, and Cochrane Library were searched from January 1, 2000, to March 31, 2026. The search terms combined MeSH/Emtree and free‐text keywords across the four domains. The full search strings are provided in Appendix S1.

### 2.3 Eligibility Criteria

The inclusion criteria were original research, systematic reviews, case series, or expert consensus in English‐language peer‐reviewed journals with complete bibliographic data. The exclusion criteria were conference abstracts, non‐peer‐reviewed commentaries, body‐contouring‐only studies, duplicates, and unverifiable publications.

### 2.4 Study Selection and Data Extraction

**FIGURE 1:**
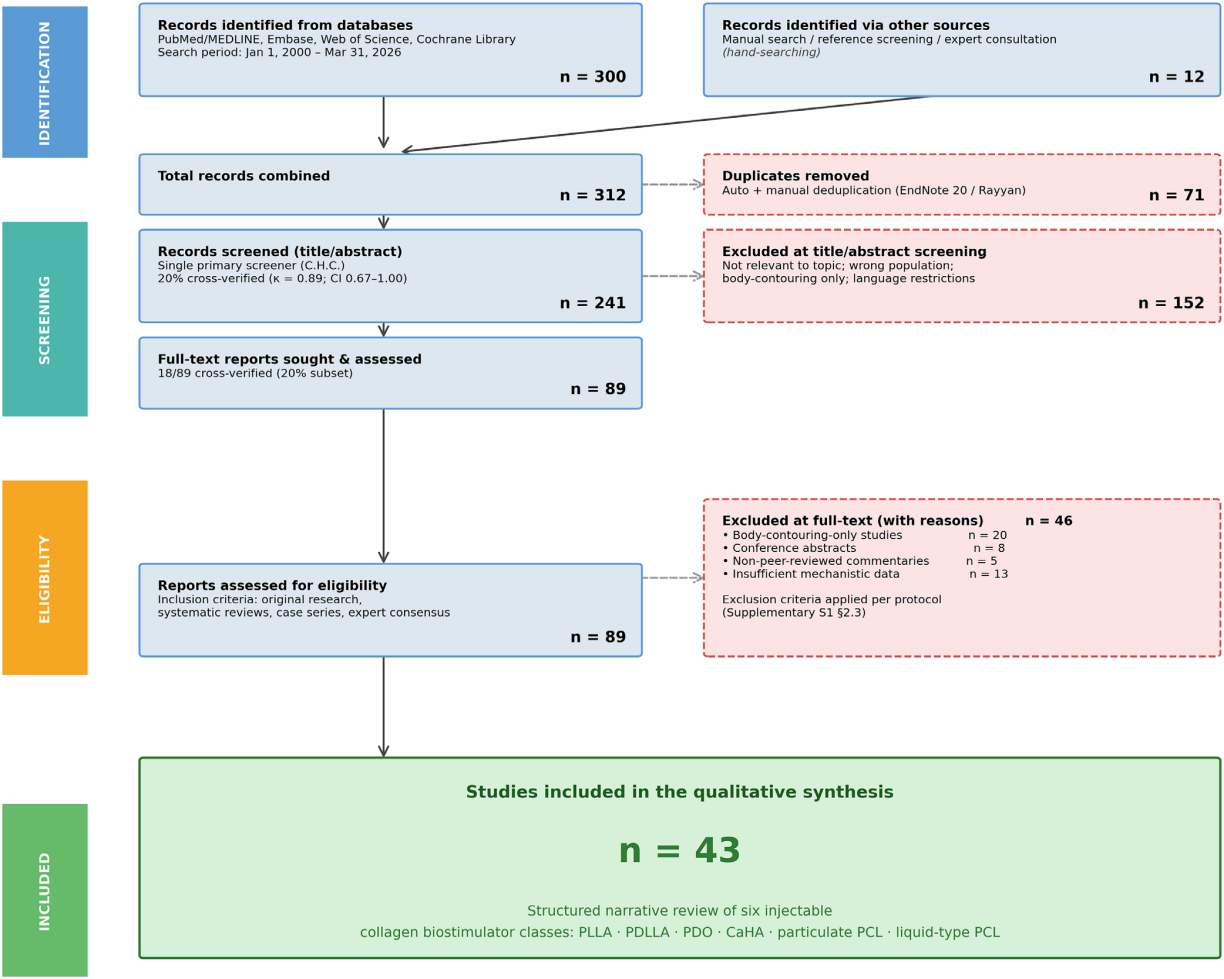
PRISMA‐informed study selection flow diagram. Of 312 total records (300 database + 12 manual), 71 duplicates removed, 152 excluded at title/abstract. Of 89 full‐text reports, 46 excluded (body‐contouring *n* = 20; conference abstracts *n* = 8; non‐peer‐reviewed *n* = 5; insufficient data *n* = 13), yielding 43 studies.

Records were exported to EndNote 20 and Rayyan software programs. Two additional members cross‐verified a random 20% subset (*n* = 18 of 89 full‐text records; Cohen's *κ* = 0.89, 95% confidence interval [CI] 0.67–1.00). However, this estimate has limited precision because the cross‐verification covered only approximately 20% of full‐text records; the lower bound of the confidence interval (0.67) indicates that true agreement could be as low as substantial, and results should be interpreted accordingly. Forty‐three studies met the final inclusion criteria (Figure 1).

## 3 Results and Discussion

### 3.1 Mechanotransduction Pathways Engaged by Biostimulator Scaffolds

**FIGURE 2:**
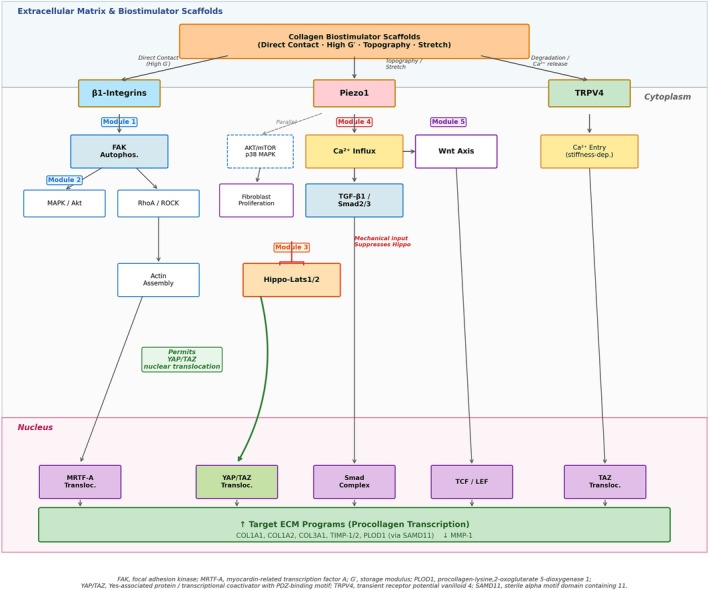
Core mechanotransduction signaling pathways engaged by collagen biostimulator scaffolds. Five interconnected modules are shown: ① Integrin–FAK–RhoA/ROCK, ② MAPK, ③ Hippo/YAP–TAZ, ④ Piezo1–Ca^2+^–TGF‐β and TRPV4, and ⑤ Wnt/β‐catenin. The “Permits” arrow from the Hippo module to YAP/TAZ indicates that suppression of Lats1/2 kinase activity releases YAP/TAZ from cytoplasmic sequestration, permitting nuclear translocation.

**FIGURE 3:**
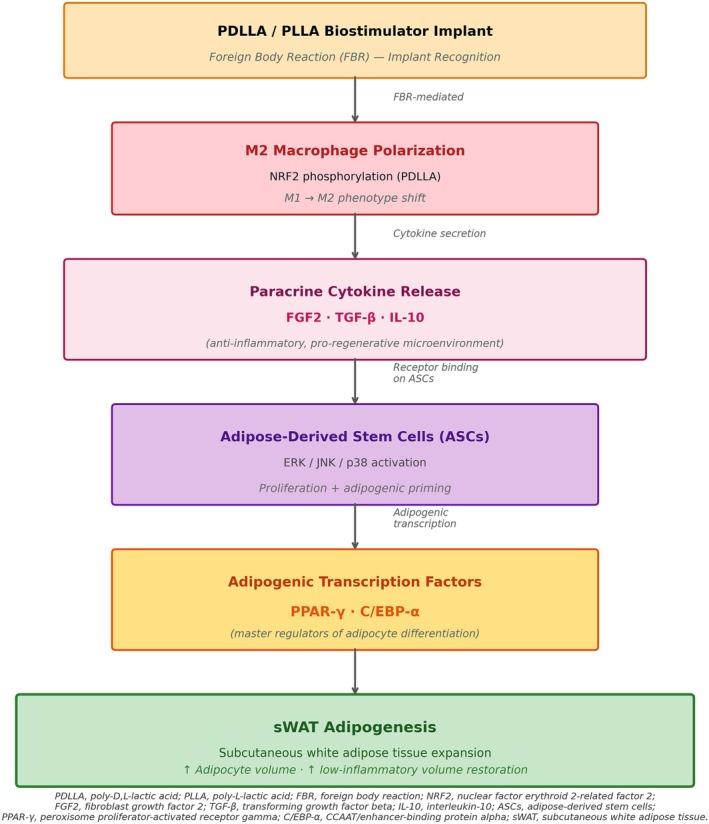
Parallel paracrine/adipogenic axis. This pathway, primarily associated with PDLLA and PLLA, operates through M2 macrophage polarization and ASC activation to promote sWAT adipogenesis. This pathway is distinct from the core mechanotransduction circuitry shown in Figure 2, although it may interact with it at the level of FGF2/TGF‐β secretion.

Five interconnected pathway modules constitute the mechanotransduction circuitry relevant to biostimulator‐driven neocollagenesis (Figure 2). First, β1‐integrins bind to extracellular matrix (ECM) proteins, triggering FAK autophosphorylation and downstream MAPK/Akt signaling [9, 10]. Second, RhoA/ROCK drives actin assembly and MRTF‐A‐mediated myofibroblast differentiation, while Rac1/Cdc42 supports cell migration [10, 11, 19, 20, 21]. Third, Hippo‐Lats1/2 suppression permits YAP/TAZ nuclear translocation (i.e., Lats1/2 inactivation releases YAP/TAZ from cytoplasmic sequestration) [11, 12, 13, 22]. Fourth, Piezo1 permits Ca^2+^ influx driving TGF‐β1/Smad2/3 signaling; TRPV4 provides additional Ca^2+^ entry, functioning as an independent mechanosensitive channel that promotes TAZ nuclear translocation in a stiffness‐dependent manner [12, 23, 24, 25]. Fifth, a Piezo1–Wnt axis activates TCF/LEF‐mediated ECM programs [23, 26]. PLLA, CaHA (via TRPV4), particulate PCL, and liquid‐type PCL each engage these mechanosensitive channels, though through distinct upstream triggers and with varying levels of direct evidence. Convergent effectors upregulate COL1A1, COL1A2, COL3A1, TIMP‐1/2, and—based on preliminary evidence—PLOD1, while suppressing MMP‐1 [13, 22, 24, 27]. A separate paracrine/adipogenic axis, primarily associated with PDLLA and PLLA, is depicted in Figure 3. Although the Piezo1–Wnt axis (Module 5) is a recognized component of general fibroblast mechanotransduction, current literature has not yet directly mapped this specific pathway to any of the commercially available biostimulator classes discussed herein.

### 3.2 Comparative Profiles of Collagen Biostimulators

#### 3.2.1 Poly‐L‐Lactic Acid

PLLA (e.g., Sculptra, Galderma) is a semi‐crystalline homopolymer presenting as irregular microflakes (2–150 μm) with tissue persistence exceeding 24 months [28]. Mechanobiologically, PLLA engages β1‐integrin modulation and FAK/MAPK/Akt activation with TGF‐β/Smad signaling [24]. Importantly, PLLA‐induced alterations in ECM stiffness activate Piezo1 mechanosensitive channels, which upregulate the AKT/mTOR and p38 MAPK pathways to promote fibroblast proliferation; notably, Piezo1‐mediated Ca^2+^ signaling may promote collagen synthesis independently of YAP/TAZ nuclear translocation, representing a parallel rather than sequential downstream event [24]. Furthermore, PLLA polarizes macrophages toward the M2 phenotype, increasing TGF‐β and IL‐10 secretion; this microenvironment stimulates myofibroblast differentiation and upregulates TIMP‐1 to protect the newly formed matrix [29]. These findings indicate that PLLA engages both FBR‐dependent (M2/TGF‐β) and FBR‐independent (Piezo1–AKT/mTOR) mechanotransduction arms. However, its irregular morphology and heavy FBR reliance pose the highest reported nodule risk among biostimulators [30].

#### 3.2.2 Poly‐D,L‐Lactic Acid

PDLLA (e.g., Juvelook, VAIM Co.) is a fully amorphous copolymer that forms porous spherical microparticles (20–100 μm) with in vitro degradation in 10–18 months [28, 31]. Unlike solid PLLA, PDLLA features a multi‐porous reticular internal architecture that facilitates inside‐out hydrolysis, yielding a less aggressive acidic microenvironment. PDLLA appears to enhance NRF2 phosphorylation, driving M2 macrophage polarization and FGF2/TGF‐β secretion [31, 32]. While PDLLA promotes M2 macrophage polarization, this process is a downstream consequence of multiple complex signaling cascades. Therefore, inferring the direct engagement of specific mechanosensors such as Piezo1 without receptor‐level evidence is unsupported, which limits the mechanistic certainty of PDLLA's pathway assignment. FGF2 activates ERK/JNK/p38 pathways in adipose‐derived stem cells (ASCs) to restore their proliferative capacity. In preclinical models, PDLLA‐stimulated macrophages upregulate adipogenic transcription factors (PPAR‐γ, C/EBP‐α) in ASCs, expanding subcutaneous white adipose tissue (sWAT) in a low‐inflammatory state [32]. This dual collagen‐adipogenic regenerative profile is potentially relevant to the fat‐depleted state seen in GLP‐1 RA–associated facial aging, although the evidence base is limited to preclinical rodent data from a single research group, and direct mechanoreceptor‐level evidence in PDLLA‐treated tissue remains absent. Nodule risk is lower than that of PLLA [31].

#### 3.2.3 Polydioxanone (Extrapolated Mechanistic Evidence)

PDO is a synthetic absorbable polyester (absorption 4–6 months) available as threads (e.g., Ultracol, Ultra V Co.) and injectable microparticles [33]. PDO is included in this comparative review because of its widespread clinical use in facial rejuvenation; however, dedicated molecular pathway studies in PDO‐treated tissue are absent. PDO threads deliver a direct mechanical stretch along linear insertion vectors, and the resulting tissue remodeling is consistent with TGF‐β/Smad‐mediated COL1A1 upregulation and RhoA/ROCK activation based on general mechanical‐tension principles, but these pathway assignments represent extrapolations rather than directly demonstrated PDO‐specific mechanisms. PDO microparticles (20–50 μm) provide a more distributed scaffold for fibroblast engagement. Regardless of the format, rapid biodegradation positions PDO adjunctively. Definitive mechanotransduction pathway assignments for PDO require dedicated in vitro and in vivo studies.

#### 3.2.4 Calcium Hydroxyapatite

CaHA (e.g., Radiesse, Merz Pharmaceuticals) comprises smooth ceramic microspheres (25–45 μm) in CMC gel, exhibiting the highest G′ among injectable biostimulators [16, 34]. In the collapsed tensional integrity of GLP‐1 RA–associated facial aging, this extreme stiffness mismatch creates a robust mechanical anchor. Direct mechanotransduction via fibroblast–microsphere contact engages β1‐integrin focal adhesion complexes, triggering FAK autophosphorylation and downstream RhoA/ROCK‐mediated YAP/TAZ nuclear translocation [22, 35]. Only fibroblasts in direct physical contact demonstrate significant collagen upregulation [35]. Additionally, Ca^2+^ ions released during biodegradation locally activate TRPV4 channels; TRPV4 has been independently shown to promote TAZ nuclear translocation in a stiffness‐dependent manner, amplifying calcium signaling independent of macrophage infiltration [25]. Histologically, significant Collagen III increases are observed at 4–9 months, with Collagen I accumulation by 9 months post‐injection [36]. This minimal FBR dependency and direct mechanotransduction profile support CaHA as a deep‐plane structural scaffold, particularly in Fitzpatrick IV–VI skin types, though its irreversibility poses significant clinical considerations. Because GLP‐1 RA users may experience dynamic weight fluctuations or facial fat rebound if the medication is discontinued, the inability to dissolve CaHA requires meticulous patient selection, ideally reserving its use for patients whose target weights have firmly stabilized [34, 36].

#### 3.2.5 Particulate Polycaprolactone

Particulate PCL (e.g., Ellansé, Sinclair Pharma) is a semi‐crystalline polyester with microspheres (25–50 μm) in a CMC gel, persisting for > 12 months. It activates a dual system: RhoA/ROCK‐mediated YAP/TAZ translocation and Piezo1‐mediated topographic sensing [9, 11, 12, 23]. Preliminary evidence from a single study suggests particulate PCL activates the SAMD11–PLOD1 axis; this finding has not yet been independently replicated and has not been confirmed in liquid‐type PCL formulations [27]. However, the focal, contact‐dependent activation field of particulate PCL inherently limits its utility as a sole scaffold in diffuse, pan‐facial presentations [28, 35].

#### 3.2.6 Liquid‐Type Polycaprolactone

Liquid‐type PCL (e.g., GOURI, DEXLEVO Inc.) is a fully solubilized, particle‐free injectable PCL manufactured via CESABP technology, eliminating risks of particle aggregation, needle occlusion, and FBGC formation [37, 38, 39, 40].

Importantly, the published evidence base for liquid‐type PCL remains limited in scale, duration, and investigator diversity, with overlapping author groups across multiple studies. Mechanotransduction claims—particularly broader‐field Piezo1/YAP/TAZ engagement—therefore remain hypothesized and await independent replication by groups without commercial relationships with the manufacturer.

**FIGURE 4:**
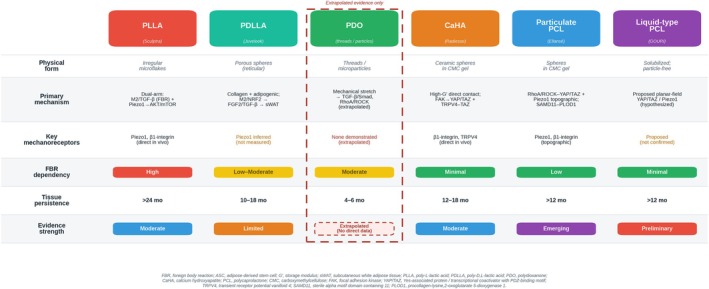
Comparative mechanotransduction profiles of six collagen biostimulator classes. This visual summary details the distinct primary mechanisms, foreign body reaction (FBR) dependencies, tissue persistence, and the strength of supporting evidence for each agent. Agents whose pathway assignments rest solely on extrapolation—most notably polydioxanone (PDO)—are visually demarcated with a dashed border and an “Extrapolated (No direct data)” evidence descriptor, distinguishing them from agents supported by direct in vivo mechanoreceptor evidence (PLLA, CaHA).

With these limitations in mind, the manufacturer proposes that liquid‐type PCL forms a three‐dimensional hydrogel meshwork upon injection, hypothetically delivering planar rather than focal tension across a broader dermal field than particulate PCL. Several preclinical and clinical studies have been reported [38, 39, 40, 41, 42, 43], including a rat model demonstrating neocollagenesis without FBR [40], a randomized split‐face trial [39], a midface pilot study [42], and case reports [38, 43]. A multicenter case series documented rare long‐term bruising attributed to pigment entrapment within the scaffold [41]; however, this adverse‐event observation does not constitute mechanistic evidence and should not be interpreted as supporting the proposed mechanism of action. A comparative summary of the mechanotransduction profiles and the strength of supporting evidence for each biostimulator class is presented in Figure 4.

### 3.3 Subgroup Considerations

Fitzpatrick IV–VI patients face elevated PIH risk. The minimal FBR and direct mechanotransduction profile of CaHA afford greater outcome predictability in these patients; liquid‐type PCL also shows limited FBR according to the available data. The pronounced FBR of PLLA warrants caution [30]. Women (~70% of users [4]) typically present with diffuse pan‐facial deflation, where the dual collagen‐adipogenic profile of PDLLA may be relevant (pending clinical confirmation) and the highest G′ of CaHA provides the strongest mechanical restoration of tensional homeostasis. Men more frequently present with focal loss where deep CaHA struts may be preferred [34, 36].

### 3.4 A Conceptual Framework for Clinical Considerations

All recommendations below represent extrapolations; no studies have directly enrolled patients with GLP‐1 RA–associated facial aging (Ozempic Face). The following conceptual framework is intended as a research scaffold, not as clinical guidance.

Based on the mechanotransduction profiles described above, the degree of facial volume loss may conceptually be stratified into three tiers. In mild presentations (limited volume deflation with preserved contour), a broad‐field scaffold approach may be considered, with selective focal reinforcement. In moderate presentations (visible mid‐face deflation with early contour disruption), a combined scaffold and deep structural strut approach may be appropriate, potentially supplemented by vector realignment. In severe presentations (extensive pan‐facial volume loss with significant contour collapse), a staged, multi‐component protocol beginning with structural restoration may be warranted.

Specific numeric thresholds (e.g., BMI delta, HFUS‐measured fat loss percentages) have been proposed as a research agenda for prospective validation and are presented in Table S1. These thresholds are based on heuristic expert opinion and should not be used as clinical decision criteria until prospectively validated.

### 3.5 Limitations

First, the screening model did not meet dual‐independent criteria, and the inter‐rater reliability estimate (*κ* = 0.89) has limited precision owing to the small cross‐verification subset (~20% of records). Second, study heterogeneity precluded a formal meta‐analysis. Third, evidence for liquid‐type PCL remains limited, with restricted investigator diversity and overlapping author groups [39, 42]. Fourth, no study has enrolled patients with GLP‐1 RA–associated facial aging; all clinical inferences are extrapolations. Fifth, geographic bias may have existed. Sixth, the severity framework thresholds are based on expert opinion and require prospective validation. Seventh, the mechanotransduction evidence base is heterogeneous across agents: PLLA and CaHA have direct in vivo mechanoreceptor studies, whereas PDLLA's Piezo1 involvement remains largely inferential from M2 polarization data; PDO pathway assignments are extrapolated from general mechanical‐tension principles; the SAMD11–PLOD1 axis [27] was demonstrated for particulate PCL and has not been confirmed in liquid‐type formulations. Future studies with agent‐matched designs are needed.

### 3.6 Conclusions

GLP‐1 RA–induced facial aging represents biomechanical and metabolic compromise. HA fillers may be limited by their relatively low G′ and primarily space‐occupying mechanism. Collagen biostimulators offer a biologically plausible—though clinically unproven—class, with each exhibiting a distinct profile supported by varying levels of evidence: PLLA engages dual Piezo1–AKT/mTOR and M2‐FBR arms (moderate evidence); PDLLA potentially addresses both collagen loss and fat depletion via sWAT adipogenesis in preclinical models (limited evidence); CaHA provides the strongest direct mechanical stimulus through high G′ and TRPV4‐TAZ signaling (moderate evidence); particulate PCL activates YAP/TAZ and Piezo1 simultaneously (emerging evidence for SAMD11–PLOD1); and liquid‐type PCL proposes broader‐field engagement requiring further validation (preliminary evidence). A conceptual severity‐stratified framework is proposed but requires validation in adequately powered prospective studies enrolling GLP‐1 RA–associated facial aging patients.

## Author Contributions

Chang‐Hwan Cho: Conceptualization, Investigation, Writing – Original Draft, Visualization. Atsuko Yanagawa: Investigation, Writing – Review and Editing, Validation. Beomjoon Kim: Supervision, Writing – Review and Editing, Validation.

## Funding

This research received no specific grant from any funding agency in the public, commercial, or not‐for‐profit sectors.

## Ethics Statement

The authors confirm that the ethical policies of the journal, as noted on the journal's author guidelines page, have been adhered to. No ethical approval was required as this is a review article with no original research data.

## Consent

No patient photographs or identifiable data are included in this manuscript.

## Conflicts of Interest

The authors declare no conflicts of interest.

## Supporting information


**Table S1:** Severity‐Stratified Framework (Research Agenda for Prospective Validation).

## Data Availability

Data sharing not applicable to this article as no datasets were generated or analysed during the current study.
