# Supplementary material for: Mechanotransduction of Collagen Biostimulators: Comparative Signaling and Implications for GLP‐1 RA–Associated Facial Aging
Source: J Cosmet Dermatol. 2026 Aug 4;25(8):e71105. doi: 10.1111/jocd.71105 (PMC13438120; doi:10.1111/jocd.71105)
Supplement: Supplementary file 1 [file JOCD-25-e71105-s001.docx]

**SUPPLEMENTARY APPENDIX**

Mechanotransduction of Collagen Biostimulators: Comparative Signaling and Implications for GLP-1 RA–Associated Facial Aging

# Contents

S1. Detailed Search Strategy

S2. PRISMA-Informed Study Selection Flow (detailed yield counts)

S3. Search Yield Summary by Source

S4. Data Extraction Template

S5. Included Studies — Characteristics Summary (n = 43)

S6. Quality Assessment / Risk-of-Bias Considerations

S7. Pathway-to-Reference Mapping (Figure 2 / Figure 3 / Figure 4)

S8. Glossary of Mechanotransduction Terms

S9. List of Abbreviations

Supplementary Table S1. Severity-Stratified Framework (Research Agenda)

# S1. Detailed Search Strategy

A PRISMA-informed literature search was conducted across four major biomedical databases to identify publications addressing mechanotransduction pathways in injectable collagen biostimulators and their clinical application in glucagon-like peptide-1 receptor agonist (GLP-1 RA)–induced facial aging. This review is a structured narrative review employing PRISMA-informed search and reporting for transparency; it does not constitute a systematic review. The search period covered January 1, 2000 through March 31, 2026..

## S1.1 | Database-specific search strings

**PubMed / MEDLINE search string (executed March 31, 2026):** (("glucagon-like peptide-1"[MeSH] OR "semaglutide"[tiab] OR "tirzepatide"[tiab] OR "liraglutide"[tiab] OR "GLP-1"[tiab] OR "Ozempic face"[tiab]) AND ("facial aging"[tiab] OR "skin aging"[MeSH] OR "dermal atrophy"[tiab])) OR (("poly-L-lactic acid"[tiab] OR "poly-D,L-lactic acid"[tiab] OR "polydioxanone"[tiab] OR "calcium hydroxylapatite"[MeSH] OR "polycaprolactone"[tiab] OR "collagen biostimulator"[tiab] OR "collagen stimulator"[tiab]) AND ("mechanotransduction, cellular"[MeSH] OR "mechanotransduction"[tiab] OR "Piezo1"[tiab] OR "YAP"[tiab] OR "TAZ"[tiab] OR "TGF-beta"[MeSH] OR "integrin"[MeSH] OR "focal adhesion kinase"[MeSH] OR "RhoA"[tiab] OR "MRTF"[tiab] OR "Wnt"[tiab] OR "TRPV4"[tiab]))

**Embase search string (executed March 31, 2026):** (('glucagon like peptide 1'/exp OR 'semaglutide':ti,ab OR 'tirzepatide':ti,ab OR 'liraglutide':ti,ab OR 'GLP-1':ti,ab OR 'Ozempic face':ti,ab) AND ('facial aging':ti,ab OR 'skin aging'/exp OR 'dermal atrophy':ti,ab)) OR (('poly-L-lactic acid':ti,ab OR 'poly-D, L-lactic acid':ti,ab OR 'polydioxanone':ti,ab OR 'calcium hydroxyapatite'/exp OR 'calcium hydroxylapatite':ti,ab OR 'polycaprolactone':ti,ab OR 'collagen biostimulator':ti,ab OR 'collagen stimulator':ti,ab) AND ('mechanotransduction'/exp OR 'mechanotransduction':ti,ab OR 'Piezo1':ti,ab OR 'YAP':ti,ab OR 'TAZ':ti,ab OR 'transforming growth factor beta'/exp OR 'TGF-beta':ti,ab OR 'integrin'/exp OR 'integrin':ti,ab OR 'focal adhesion kinase 1'/exp OR 'focal adhesion kinase':ti,ab OR 'RhoA':ti,ab OR 'MRTF':ti,ab OR 'Wnt':ti,ab OR 'TRPV4':ti,ab)).

**Web of Science - Core Collection search string (executed March 31, 2026):** (TS=("glucagon-like peptide-1" OR "semaglutide" OR "tirzepatide" OR "liraglutide" OR "GLP-1" OR "Ozempic face") AND TS=("facial aging" OR "skin aging" OR "dermal atrophy")) OR (TS=("poly-L-lactic acid" OR "poly-D, L-lactic acid" OR "polydioxanone" OR "calcium hydroxylapatite" OR "polycaprolactone" OR "collagen biostimulator" OR "collagen stimulator") AND TS=("mechanotransduction, cellular" OR "mechanotransduction" OR "Piezo1" OR "YAP" OR "TAZ" OR "TGF-beta" OR "integrin" OR "focal adhesion kinase" OR "RhoA" OR "MRTF" OR "Wnt" OR "TRPV4")).

**Cochrane Library search string (executed March 31, 2026):** (([mh "Glucagon-Like Peptide 1"] OR "semaglutide":ti,ab,kw OR "tirzepatide":ti,ab,kw OR "liraglutide":ti,ab,kw OR "GLP-1":ti,ab,kw OR "Ozempic face":ti,ab,kw) AND ("facial aging":ti,ab,kw OR [mh "Skin Aging"] OR "dermal atrophy":ti,ab,kw)) OR (("poly-L-lactic acid":ti,ab,kw OR "poly-D, L-lactic acid":ti,ab,kw OR "polydioxanone":ti,ab,kw OR [mh "Durapatite"] OR "calcium hydroxylapatite":ti,ab,kw OR "polycaprolactone":ti,ab,kw OR "collagen biostimulator":ti,ab,kw OR "collagen stimulator":ti,ab,kw) AND ([mh "Mechanotransduction, Cellular"] OR "mechanotransduction":ti,ab,kw OR "Piezo1":ti,ab,kw OR "YAP":ti,ab,kw OR "TAZ":ti,ab,kw OR [mh "Transforming Growth Factor beta"] OR "TGF-beta":ti,ab,kw OR [mh "Integrins"] OR "integrin":ti,ab,kw OR [mh "Focal Adhesion Kinase 1"] OR "focal adhesion kinase":ti,ab,kw OR "RhoA":ti,ab,kw OR "MRTF":ti,ab,kw OR "Wnt":ti,ab,kw OR "TRPV4":ti,ab,kw))

## S1.2 | Screening and Data Extraction Workflow

All identified records were exported to EndNote 20 (Clarivate Analytics) for deduplication, and subsequently uploaded to Rayyan (Qatar Computing Research Institute) for collaborative screening. The primary screener (C.H.C.) conducted title/abstract screening followed by full-text assessment of all retained records. Two additional reviewers (A.Y. and B.K.) independently cross-verified a random 20% subset (n = 18 of 89 full-text records) using a pre-specified data extraction form **(provided in Section S4)**. Disagreements were resolved by consensus discussion.

Inter-rater agreement on the cross-verified subset was Cohen’s κ = 0.89 (95% CI 0.67–1.00). However, this estimate has limited precision because the cross-verification covered only approximately 20% of full-text records; the lower bound of the confidence interval (0.67) indicates that true agreement could be as low as substantial. The review authors explicitly acknowledge that this screening model does not meet the PRISMA dual-independent criterion for systematic reviews; accordingly, this work is positioned as a structured narrative review intended for hypothesis generation rather than for statistical pooling of effect estimates.

# S2. PRISMA-Informed Study Selection Flow

The detailed numerical flow of records through each stage is consistent with that presented in Figure 1 of the main manuscript and the original submission. Total: 312 records identified → 71 duplicates removed → 241 screened → 152 excluded at title/abstract → 89 full-text assessed (18 cross-verified) → 46 excluded at full-text → 43 studies included.

# S3. Search Yield Summary by Source

A total of 312 records were initially identified. The distribution by source before deduplication was as follows:

- **Databases (PubMed/MEDLINE, Embase, Web of Science, Cochrane Library):** 300 records
- **Other sources (Manual search / reference screening / expert consultation):** 12 records

# S4. Data Extraction Template

To ensure consistency and reproducibility during the cross-verification of full-text records, the following pre-specified data extraction template was utilized by the independent reviewers:

| **Data Domain** | **Specific Elements Extracted** |
| --- | --- |
| Study Characteristics | Author(s), Publication year, Country of origin, Study design (*in vitro, in vivo* animal, clinical trial, case series, review/consensus) |
| Biostimulator Scaffold | Agent class (PLLA, PDLLA, PDO, CaHA, Particulate PCL, Liquid-type PCL), Physical form, Rheological properties (e.g., G′) |
| Mechanotransduction Pathways | Upstream physical triggers (stiffness, topography, stretch), Engaged mechanoreceptors (Piezo1, TRPV4, β1-integrin), Intracellular signaling molecules (YAP/TAZ, FAK, RhoA/ROCK, MAPK, Smad) |
| Paracrine / FBR Profile | Macrophage polarization (M1/M2 shift), Cytokine expression (TGF-β, FGF2, IL-10), Adipogenic transcription factors (PPAR-γ, C/EBP-α), Stem cell activation (ASCs) |
| Key Outcomes | Target ECM gene upregulation (COL1A1, COL3A1, TIMP-1, PLOD1), Neocollagenesis timeframe, Tissue persistence, Safety profile |
| Clinical Relevance | Mentions of weight loss-induced facial aging, GLP-1 RA use, and structural tensional homeostasis restoration |

# S5. Included Studies — Characteristics Summary (n = 43)

Due to space constraints, the full data extraction table detailing all 43 included studies is not presented here but is available from the corresponding author upon reasonable request. Key study characteristics, extracted mechanistic pathways, and evidence strengths have been qualitatively synthesized throughout Sections 3.1 and 3.2 of the main manuscript.

# S6. Quality Assessment / Risk-of-Bias Considerations

As a structured narrative review, formal risk-of-bias tools (e.g., ROBINS-I, RoB 2.0, AMSTAR-2) were not applied to individual studies. Instead, the following qualitative appraisal framework was used to inform the strength of mechanistic claims and clinical inferences drawn in the main text. This framework directly informs the “Evidence Strength” column added to Table 1 in the revised manuscript:

| **Evidence domain** | **Strength of evidence** | **Representative studies** |
| --- | --- | --- |
| Direct in vivo human mechanotransduction data (PLLA, CaHA) | Moderate — multiple independent groups, consistent findings | *refs. 24, 29, 34, 35, 36* |
| In vitro molecular mechanism (YAP/TAZ, Piezo1, TRPV4) | Strong — foundational studies widely cited | *refs. 10, 11, 12, 22, 23, 25* |
| PDLLA adipogenic pathway (NRF2 / PPAR-γ / C/EBP-α) | Limited — primarily preclinical rodent data from a single research group; Piezo1 inferred, not directly measured | *refs. 31, 32* |
| PDO molecular pathway (direct studies) | Extrapolated (no direct data) — pathway inferences extrapolated from general mechanical-tension principles; PDO is visually demarcated in Figure 4 | *ref. 33* |
| Particulate PCL SAMD11–PLOD1 axis | Emerging — single primary study, not yet independently replicated | *ref. 27* |
| Liquid-type PCL broader-field engagement (proposed) | Preliminary — limited scale, short duration, restricted investigator diversity with overlapping author groups; mechanotransduction claims await independent replication | *refs. 38–43* |
| GLP-1 RA-specific facial aging studies | Absent — no studies directly enrolled Ozempic Face patients; all clinical inferences are extrapolations | *refs. 5, 6 (observational narratives only)* |

# S7. Pathway-to-Reference Mapping (Figure 2/ Figure 3/ Figure 4)

The pathway-to-reference mapping is consistent with the original submission, with the following structural change: Figure 2 has been split into Figure 2 (core mechanotransduction modules ①–⑤) and Figure 3 (parallel paracrine/adipogenic axis). The paracrine adipogenesis pathway (M2 polarization → ASC activation → sWAT expansion), primarily associated with PDLLA and PLLA, is now depicted separately in Figure 3 to avoid conflating mechanotransduction and paracrine signaling categories. The “Permits” arrow in Figure 2 from the Hippo module to YAP/TAZ translocation is clarified in the legend to indicate that Lats1/2 suppression releases YAP/TAZ from cytoplasmic sequestration. In addition, Figure 4 has been revised so that polydioxanone (PDO), whose pathway assignments rest solely on extrapolation from general mechanical-tension principles, is visually isolated by a dashed border and labelled “Extrapolated (No direct data),” distinguishing it from agents supported by direct in vivo mechanoreceptor evidence (PLLA, CaHA).

# Supplementary Table S1. Severity-Stratified Framework (Research Agenda for Prospective Validation)

**Important:** The numeric thresholds presented below are based on heuristic expert opinion and have not been clinically validated. They are presented solely as a research agenda to guide the design of future prospective studies. These thresholds should not be used as clinical decision criteria. No studies have directly enrolled patients with GLP-1 RA–associated facial aging.

| **Severity (validation pending)** | **Proposed numeric correlates (expert opinion)** | **Primary agent** | **Supporting** |
| --- | --- | --- | --- |
| Mild | BMI Δ<5; HFUS loss <15% | Broad-field scaffold (e.g., liquid-type PCL) | Selective focal CaHA |
| Moderate | BMI Δ 5–10; HFUS 15–30% | Scaffold + deep CaHA struts | PDO for vector realignment |
| Severe | BMI Δ>10; HFUS >30% | Sequential CaHA then scaffold; PLLA in cushioned compartments | Multi-component; staged |

*BMI, body mass index; HFUS, high-frequency ultrasound. All thresholds represent unvalidated expert opinion requiring prospective confirmation in GLP-1 RA–associated facial aging cohorts.*

# S8. Glossary of Mechanotransduction Terms

The glossary remains unchanged from the initial submission. Key terms defined include: α-SMA, β-catenin, CCN2 (CTGF), FAK, foreign body reaction (FBR), G′ (storage modulus), Hippo pathway, integrin β1, MAPK, MRTF-A, myofibroblast, PIEZO1, PLOD1, RhoA/ROCK, SAMD11, Smad2/3, SRF, TCF/LEF, TEAD, TRPV4, and YAP/TAZ.

# S9. List of Abbreviations

The abbreviation list remains unchanged from the initial submission.

*— END OF SUPPLEMENTARY APPENDIX —*
